# Investigating the Relationship between Fingerprint Pattern and Development of Oral Squamous Cell Carcinoma

**DOI:** 10.30476/DENTJODS.2021.87173.1240

**Published:** 2022-06

**Authors:** Arghavan Tonkaboni, Mahdi Etemadian, Soheila Manifar, Mohammad Shirkhoda, Jaber Gharehdaghi, Mohammad Javad Kharazi Fard

**Affiliations:** 1 Dept. of Oral Medicine, School of Dentistry, Tehran University of Medical Sciences, Tehran, Iran; 2 Grupo De Investigación En Patología Oral Médico Quirúrgica, University of Santiago de Compostela, Spain; 3 Student, School of Dentistry, Tehran University of Medical Sciences, International Campus, Tehran, Iran; 4 Dept. of Oral Medicine, Imam Khomeini Hospital, Tehran University of Medical Sciences, Tehran, Iran; 5 Dept. of Oncosurgery, Cancer Institute, Tehran University of Medical Sciences, Tehran, Iran; 6 Legal Medicine Research Center, Legal Medicine Organization, Tehran, Iran; 7 Dental Research Center, Dentistry Research Institute, Tehran University of Medical Sciences, Tehran, Iran

**Keywords:** Carcinoma, Squamous cell, Mouth neoplasm, Dermatoglyphic, Epigenomics

## Abstract

**Statement of the Problem:**

Oral squamous cell carcinoma (OSCC) constitutes more than 90% of oral malignancies. The main risk factors of OSCC include cigarette smoking and alcohol. However, since not all smokers or alcohol drinkers develop this disease, other factors have also been suggested including genetic characteristics of every person to be implicated in the probability of developing OSCC.

**Purpose:**

Our aim in this study is to investigate the possible relationship between fingerprint patterns and the probability of developing OSCC.

**Materials and Method:**

In a cross sectional study, we had 140 patients in 2 groups as OSCC and cancer free. Fingerprints were recorded by fingerprint scanner device. The fingerprint patterns were categorized into three major groups and four subgroups. Groups were tested by chi-square.

**Results:**

The relationship between the main fingerprint patterns and incidence of OSCC became significant (*p*= 0.037). The frequency of the main pattern of Arch was significantly higher in the experimental group than in the control group (*p*< 0.05). Considering the main patterns of Loop and Whorl, no significant difference existed between the two groups. Furthermore, the frequency of subtype patterns of Double Whorl and Central Pocket Whorl was significantly higher in the control group than in the experimental group (*p*< 0.05).

**Conclusion:**

Since dermatoglyphics is contingent upon genetic variations, fingerprint can be used for investigating the susceptibility of people in developing different diseases, though further studies are required in this regard. This method is in no way a substitute for gold standard methods for diagnosis.

## Introduction

Oral cancer is the sixth common cancer in the world and one of the 10 common causes of mortality. Oral squamous cell carcinoma (OSCC) constitutes more than 90% of oral malignancies [ 1 - 2 ]. Its etiology is multifactorial and there is no single factor known for its development, whereby both genetic and epigenetic factors can be involved. The main risk factors of OSCC include cigarette smoking and alcohol. However, since not all smokers or alcohol drinkers develop this disease, other factors have also been suggested including genetic characteristics of every person to be implicated in the probability of developing OSCC [ 3 - 7 ]. 

Dermatoglyphics studies the skin lines present in the palm and sole as well as tip of the fingers and toes [ 8 - 9 ]. A multi-gene system controls the creation and development of skin lines. Since the genes participating in controlling skin lines have not been found completely, and considering the concurrency of formation of skin lines with that of many important tissues of the body, it is possible that the dermatoglyphic patterns could highlight the genetic makeup of a person and the possibility of developing some diseases with a genetic basis. Extensive research has examined the skin lines in patients with tuberculosis, insulin-dependent diabetes, breast cancer, asthma, leukemia, hypothyroidism, coronary artery disease, schizophrenia, Down syndrome, Klein Falter syndrome, Turner syndrome, Trisomy 18, Alzheimer's, rubella, rheumatoid arthritis, and psychological disease. In many of these studies, a significant difference has been reported between patient and control groups. In different areas of dentistry, dermatoglyphic patterns have been investigated considering the shared origin of oral tissues and skin lines, being the ectoderm layer, as well as the concurrency of the formation of oral tissues and these lines beginning from the fourth embryonic week up to 13th embryonic week. The relationship between skin lines and different diseases has been investigated including dental caries, bruxism, periodontal diseases, cleft lips, oral submucosal fibrosis, oral leukoplakia, mesiodens and skeletal malocclusions [ 8 - 16 ]. 

Our aim is to investigate the possible relationship between fingerprint patterns and the probability of developing OSCC.

## Materials and Method

We performed a cross sectional study on 140 patients referring to Cancer Institute of Imam Khomeini Hospital, Tehran. Patients were categorized into two 70-subject. In the first group, 36 males and 34 females with OSCC and in the second group, 36 males, and 34 females with no oral lesion were present. They were matched against the experimental group in terms of gender, age, history of smoking, and alcohol consumption. The sampling method was convenience sampling based on the inclusion criteria. 

The inclusion criteria in the experimental group were defined as the patient should have had OSCC as confirmed by a pathologist, while in the control group, the subjects should have been healthy with no history of oral lesion so far. In both experimental and control groups; the subjects should have been Iranian. The exclusion criteria were defined as impossibility of recording the fingerprint of a subject because of amputation or organ defect, and those with any systemic disease.

Patients were informed in detail about the study and their informed consent was obtained to conduct the study. This study was approved by the Ethics Committee of Tehran University of Medical Sciences (IR.TUMS. DENTISTRY.REC.1397.061). Initially, basic information including name, surname, gender, age, histopathology diagnosis and site of lesion (in the patient group), history of smoking, history of alcohol consumption, and history of developing systemic disease was completed. All patients cleaned their fingertips by water and soap in order to remove any contamination and dirt. Once the cleanness of hands was ensured, the fingerprints were recorded sequentially under the supervision of an operator by FS80H fingerprint scanner device (Futronic Co. Hong Kong). Thereafter, the recorded fingerprints were provided to forensic medicine specialists to analyze and determine the pattern of each finger. The fingerprint patterns were categorized into three major groups as Whorl, Loop, and Arch along with subgroups of Simple Arch, Tented Arch, Radial Loop, Ulnar Loop, Simple Whorl, Double Whorl, Central Pocket Whorl, and Accidental Whorl.

In order to compare the frequency of the fingerprints in the experimental and control groups, chi-square test was used. Next, through calculating the odds ratio related to each of the fingerprint patterns in both patient and healthy subjects, and if confidence interval of 95% of odds ratio did not confirm the assumption of equality of these ratios, a significant difference about that type of fingerprint was considered between the healthy and patient groups. All the above analyses were performed using SPSS Software (Statistical Package for Social Science) version 25. A *p* Value less than 0.05 were considered statistically significant. 

## Results

In this study, with regard to the inclusion and exclusion criteria, 70 patients with OSCC referring to cancer Institute of Imam Khomeini Hospital, Tehran were included in the experimental group consisting of 36 males and 34 squamous cell carcinoma of buccal mucosa and gingiva, 4% squamous cell carcinoma of the lip, and 3% squamous cell carcinoma of the palate. On the other hand, in the control group, 70 healthy people with no history of OSCC and no observation of suspicious lesion in routine dentistry examinations including 36 males and 34 females with the mean age of 52±11.9 were included. The participants in this group were individually matched against the control group in terms of age (with the maximum age difference of two years), gender, history of smoking, and history of alcohol consumption.

Based on the history taken from the subjects in the present study, 23, 6 and 3% of cases in each of the groups reported a history of cigarette smoking, alcohol consumption and concurrent alcohol and cigarette use, respectively.

Based on the analyses, in both experimental and control groups, the maximum frequency belonged to Ulnar loop (43% in the experimental group and 45.1% in the control group) followed by simple Whorl (40.7% in the experimental group and 36.7% in the control group). The minimum frequency of fingerprint patterns in the experimental group was related to Central pocket loop (2.4%) while in the control group it was tented arch (2.1%).

The frequency of the major types of the fingerprint patterns in all fingers in both experimental and control groups are presented in Table 1. Based on the comparison, the relationship between the main fingerprint patterns and incidence of OSCC became significant. The frequency of the main pattern of Arch was significantly higher in the experimental group than in the control group (*p*< 0.05). Concerning the main patterns of Loop and Whorl, no significant difference existed between the two groups (Table 1).

**Table 1:** Comparison of difference in prevalence of the main fingerprint patterns in different study groups in all fingers

Fingerprint Pattern	Group 1	Group 2	Odds Ratio	*p* Value	Significance
Arch	47 (6.7%)	35 (5%)	1.34	0.037	Significant
Loop	325 (46.4%)	337 (48.1%)	0.96	0.241	Not Significant
Whorl	328 (46.8%)	328 (46.9%)	0.99	0.377	Not Significant

The frequency of the subtypes of the fingerprint patterns in all fingers in both experimental and control groups have been reported in Table 2. Based on the comparison, the relationship between subtypes of fingerprint patterns and OSCC became significant; the frequency of the subtype of Tented arch was significantly higher in the experimental group than in the control group (*p*< 0.05). Furthermore, the frequency of subtype patterns of Double Whorl and Central Pocket Whorl was significantly higher in the control group than in the experimental group (*p*< 0.05) (Table 2).

**Table 2:** Comparison of difference in prevalence of the side fingerprint patterns in different study groups in all fingers

Fingerprint Pattern	Group 1	Group 2	Odds Ratio	*p* Value	Significance
Simple Arch	21 (3%)	20 (2.9%)	1.03	0.431	Not Significant
Tented Arch	26 (3.7%)	15 (2.1%)	1.76	0.027	Significant
Radial Loop	24 (3.4%)	21 (3%)	1.13	0.129	Not Significant
Ulnar Loop	301 (43%)	316 (45.1%)	0.95	0.451	Not Significant
Simple Whorl	285 (40.7%)	257 (36.7%)	1.1	0.390	Not Significant
Double Whorl	26 (3.7%)	32 (4.6%)	0.8	0.047	Significant
Central Pocket Whorl	17 (2.4%)	39 (5.6%)	0.42	0.039	Significant
Accidental Whorl	0	0	---	---	---

The frequency of the major types of fingerprint patterns for every finger in both the experimental and control groups has been reported in Tables 3a and 3b. According to the comparison, the frequency of the major type of arch was significantly higher in the middle finger of the left hand (odds ratio=2.24, *p*< 0.05), middle finger of the right hand (odds ratio=2.65, *p*< 0.05) and thumb of the left hand (odds ratio=2, *p*< 0.05), in the experimental group as compared to the control group.

**Table 3:** a: Prevalence of the main fingerprint pattern in different study groups in left hand

		Experiment			Control		
		Arch	Loop	Whorl	Arch	Loop	Whorl
Little finger	Frequency	1	46	23	2	48	20
	Frequency percentage	1.4%	65.7%	32.9%	2.8%	68.6%	28.6%
Ring finger	Frequency	3	29	38	3	25	42
	Frequency percentage	4.3%	41.4%	54.3%	4.3%	35.7%	60%
Middle finger	Frequency	9	35	26	4	41	25
	Frequency percentage	12.8%	50%	37.2%	5.7%	58.6%	35.7%
Index finger	Frequency	9	24	37	7	30	33
	Frequency percentage	12.8%	34.3%	52.9%	10%	42.8%	47.2%
Thumb	Frequency	6	24	40	3	28	39
	Frequency percentage	8.6%	34.3%	57.1%	4.3%	40%	55.7%
**b:** Prevalence of the main fingerprint patterns in different study groups in right hand							
		**Experiment**			**Control**		
		**Arch**	**Loop**	**Whorl**	**Arch**	**Loop**	**Whorl**
Little finger	Frequency	0	50	20	1	43	26
	Frequency percentage	0%	71.5%	28.5%	1.4%	61.4%	37.2%
Ring finger	Frequency	2	23	45	3	23	44
	Frequency percentage	2.8%	32.9%	64.3%	4.3%	32.9%	62.8%
Middle finger	Frequency	8	42	20	3	47	20
	Frequency percentage	11.4%	60%	28.6%	4.3%	67.1%	28.6%
Index finger	Frequency	6	29	35	9	23	38
	Frequency percentage	8.6%	41.4%	50%	12.8%	32.9%	54.3%
Thumb	Frequency	3	23	44	0	29	41
	Frequency percentage	4.3%	32.9%	62.8%	0%	41.4%	58.6%

Furthermore, the frequency of the subtypes of fingerprint patterns for every finger in the experimental and control groups is presented in Tables 4a and 4b and compared with other, with odds ratio calculated either. Based on the comparison, the frequency of the subtype of simple arch in the thumb of the left hand (odds ratio= 2, *p*< 0.05), Tented arch in the middle finger of the left hand (odds ratio=2.96, *p*< 0.05) and middle finger of the right hand (odds ratio=7, *p*< 0.05) has been significantly higher in the experiment a group than in the control group. On the other hand, the frequency of the subtype of double whorl in the index finger of the right hand (odds ratio=0.4, *p*< 0.05) and Central pocket whorl in the ring finger of the left hand (odds ratio=0.5, *p*< 0.05) and ring finger of the right hand (odds ratio=0.2, *p*< 0.05) and little finger of the right hand (odds ratio=0.57, *p*< 0.05) has been significantly higher in the control group than in the experimental group. (Table 3a, Table 3b, Table 4a, Table 4b).

**Table 4:** a: Prevalence of the side fingerprint patterns in experiment group in each fingers

	Simple Arch	Tented Arch	Ulnar Loop	Radial Loop	Simple Whorl	Double Whorl	Central Pocket Whorl
Little finger of the left hand	0	1	46	0	21	0	2
	0%	1.4%	65.7%	0%	30%	0%	2.9%
Ring finger of the left hand	2	1	28	1	35	0	3
	2.9%	1.4%	40%	1.4%	50%	0%	4.3%
Middle finger of the left hand	3	6	34	1	24	2	0
	4.3%	8.6%	48.6%	1.4%	34.3%	2.9%	0%
Index finger of the left hand	4	5	17	7	28	6	3
	5.7%	7.1%	24.3%	10%	40%	8.6%	4.3%
Thumb of the left hand	6	0	24	0	31	8	1
	8.6%	0%	34.3%	0%	44.3%	11.4%	1.4%
Thumb of the right hand	3	0	22	1	39	5	0
	4.3%	0%	31.4%	1.4%	55.7%	7.1%	0%
Index finger of the right hand	1	5	19	10	31	2	2
	1.4%	7.1%	27.1%	14.3%	44.3%	2.9%	2.9%
Middle finger of the right hand	1	7	41	1	19	1	0
	1.4%	10%	58.6%	1.4%	27.1%	1.4%	0%
Ring finger of the right hand	1	1	22	1	42	1	2
	1.4%	1.4%	31.4%	1.4%	60%	1.4%	2.9%
Little finger of the right hand	0	0	48	2	15	1	4
	0%	0%	68.6%	2.9%	21.4%	1.4%	5.7%
**b:** Prevalence of the side fingerprint patterns in control group in each fingers							
	**Simple Arch**	**Tented Arch**	**Ulnar Loop**	**Radial Loop**	**Simple Whorl**	**Double Whorl**	**Central Pocket Whorl**
Little finger of the left hand	1	1	48	0	12	4	4
	1.4%	1.4%	68.6%	0%	17.1%	5.7%	5.7%
Ring finger of the left hand	1	2	25	0	33	3	6
	1.4%	2.9%	35.7%	0%	47.1%	4.3%	8.6%
Middle finger of the left hand	2	2	41	0	21	2	2
	2.9%	2.9%	58.6%	0%	30%	2.9%	2.9%
Index finger of the left hand	3	4	20	10	27	3	3
	4.3%	5.7%	28.6%	14.3%	38.6%	4.3%	4.3%
Thumb of the left hand	1	2	28	0	30	9	0
	1.4%	2.9%	40%	0%	42.9%	12.9%	0%
Thumb of the right hand	0	0	27	2	34	5	2
	0%	0%	38.6%	2.9%	48.6%	7.1%	2.9%
Index finger of the right hand	8	1	16	7	31	5	2
	11.4%	1.4%	22.9%	10%	44.3%	7.1%	2.9%
Middle finger of the right hand	2	1	46	1	17	0	3
	2.9%	1.4%	65.7%	1.4%	24.3%	0%	4.3%
Ring finger of the right hand	1	2	23	0	34	0	10
	1.4%	2.9%	32.9%	0%	48.6%	0%	14.3%
Little finger of the right hand	1	0	42	1	18	1	7
	1.4%	0%	60%	1.4%	25.7%	1.4%	10%

## Discussion

Regarding the increasing rate of alcohol and cigarette consumption in today's societies, the prevalence of oral cancer is growing and may change into a major public health concern [ 3 , 13 ].

Some studies have reported that hereditary factors are also involved in incidence of OSCC, as it has been observed that individuals whose first-degree relatives had OSCC are at higher risk of developing the cancer. Moreover, it has been found that not all cigarette smokers or alcohol consumers (as the main risk factors of OSCC) develop OSCC and not all individuals who develop OSCC either smoke cigarette or consume alcohol [ 3 , 13 ]. Hence, it can be concluded that the genetic characteristics of every person can also be involved in the possibility of developing OSCC [ 3 , 13 , 17 ].

Skin lines have special characteristics that make them important. These lines have found significance in both identity detection and in medical as well as genetic studies. These lines are formed during the embryonic period and under the influence of different genes lying on various chromosomes. Apart from their size, these lines do not change throughout the life. Dermatoglyphics is a science dealing with investigating these lines both quantitatively and qualitatively and can be used as a genetic marker in diseases [ 12 - 13 , 17 - 18 ].

Since genetics plays a significant role in all cancers, in different studies, the relationship between qualitative and quantitative analyses of fingerprint and cancers such as thyroid, breast, prostate and cervix has been investigated, in many of which a significant relationship has been found [ 3 , 13 , 19 ]. The present study deals with investigating a possible relationship between fingerprint patterns and the probability of developing OSCC, so that through dermatoglyphic as a facility, the individuals who are genetically highly susceptible to developing OSCC would be identified.

The differentiating point of this study is consideration and comparison of subtypes of fingerprint and its evaluation in all fingers. In some studies, the frequency of the main pattern of Arch was significantly higher in the patient group with oral cancer than in the control group, which is in line with the present study [ 3 , 20 - 22 ].

In some studies, the frequency of Whorl main pattern was higher in the control group; since the subtypes of Double Whorl and Central Pocket Whorl are of Whorl type, it can be stated that the results were congruent with ours [ 3 , 17 , 22 ].

Based on the results of Venkatesh *et al*. [ 18 ] as well as Kadam *et al*. [ 23 ], the frequency of the main patterns of Arch and Loop was significantly higher in the experimental group than in the control group. In the present study, the frequency of loop pattern did not differ significantly between the control and experimental groups. In this regard, it is not in line with previous studies, but the results related to the Arch fingerprint pattern in the present study are consistent with others' findings [ 18 , 23 ].

Gupta *et al*. [ 13 ] compared the frequency of Arch, Ulnar Loop, Radial Loop, Simple Whorl, and Complex Whorl patterns and stated that the frequency of Arch and Ulnar Loop patterns was higher in the patient group, while the frequency of Simple Whorl was higher in the control group. The results related to the Arch fingerprint pattern in that study and ours are the same; however, in the present study, the results related to the analysis of Ulnar Loop and Simple Whorl was not significant [ 13 ].

In the study by Vinothini *et al*.[ 17 ] as well as David *et al*. [ 20 ], the frequency of the Loop major pattern was significantly higher in the patient group than in the control; the results of these studies were incongruent with ours [ 17 , 20 ].

Based on the results of Ganvir *et al*. [ 24 ] as well as Patil *et al*. [ 25 ], the frequency of Whorl major pattern in the experimental group was significantly higher than in the control; on the other hand, the frequency of Loop major pattern in the study by Ganvir and Ulnar Loop soft pattern in the study by Patil was significantly higher in the control group than in the experimental group. In the present study, the frequency of these two major types of fingerprint did not differ significantly between the two groups, which are inconsistent with these mentioned studies [ 24 - 25 ].

Based on the analyses of this study, in both experimental and control groups, the maximum frequency belongs to Ulnar Loop followed by Simple Whorl, which is in line with the findings of Ghosh *et al*. [ 26 ].

One of the reasons for the differences across these studies is the study population because it has been stated that fingerprint patterns are different across different races and communities. Since in these studies, the race of the subjects has not been mentioned, these differences can be attributed to this issue and should be considered before employing the results in clinical practice [ 25 ].

## Conclusion

Since dermatoglyphics is contingent upon genetic variations and as in most malignancies both genetic and environmental factors are involved, fingerprint can be used for investigating the susceptibility of people in developing different diseases, though further studies are required in this regard. This method is in no way a substitute for gold standard methods for diagnosis. Nevertheless, dermatoglyphic can be used as a helpful index in order to identifying susceptible populations and scheduling screening and preventive programs, which is capable to decrease the rate of OSCC.

## Conflict of Interest

None declared.
